# Post-Acute COVID-19 Symptoms, a Potential Link with Myalgic Encephalomyelitis/Chronic Fatigue Syndrome: A 6-Month Survey in a Mexican Cohort

**DOI:** 10.3390/brainsci11060760

**Published:** 2021-06-08

**Authors:** J Antonio González-Hermosillo, Jhanea Patricia Martínez-López, Sofía Antonieta Carrillo-Lampón, Dayanara Ruiz-Ojeda, Sharon Herrera-Ramírez, Luis M. Amezcua-Guerra, María del Rocío Martínez-Alvarado

**Affiliations:** 1 Department of Cardiovascular Dysautonomia, Instituto Nacional de Cardiología Ignacio Chávez, Mexico City 14080, Mexico; sincope39@yahoo.com.mx (J.A.G.-H.); jhaneaml@gmail.com (J.P.M.-L.); sofia.clampon@gmail.com (S.A.C.-L.); daayziur@gmail.com (D.R.-O.); sharon.herram@gmail.com (S.H.-R.); 2 Department of Immunology, Instituto Nacional de Cardiología Ignacio Chávez, Mexico City 14080, Mexico; lmamezcuag@gmail.com; 3 Department of Endocrinology, Instituto Nacional de Cardiología Ignacio Chávez, Mexico City 14080, Mexico

**Keywords:** fatigue, symptoms of COVID-19, Myalgic Encephalomyelitis/Chronic Fatigue Syndrome

## Abstract

The aim of this study was to describe the clinical evolution during 6 months of follow-up of adults recovered from COVID-19. We tried to determine how many met the definition of Myalgic Encephalomyelitis/Chronic Fatigue Syndrome (ME/CFS). A total of 130 patients (51.0 ± 14 years, 34.6% female) were enrolled. Symptoms were common, participants reported a median number of 9 (IQR 5–14) symptoms. Fatigue was the most common symptom (61/130; 46.9%). Patients with fatigue were older 53.9 ± 13.5 years compared with 48.5 ± 13.3 years in those without fatigue (*p* = 0.02) and had a longer length of hospital stay, 17 ± 14 days vs. 13 ± 10 days (*p* = 0.04). There was no difference in other comorbidities between patients with fatigue and those without it, and no association between COVID-19 severity and fatigue. After multivariate adjustment of all baseline clinical features, only age 40 to 50 years old was positively associated with fatigue, OR 2.5 (95% CI 1.05–6.05) *p* = 0.03. In our survey, only 17 (13%) patients met the Institute of Medicine’s criteria for “systemic exertion intolerance disease,” the new name of ME/CFS. In conclusion, in some patients, the features of post-acute COVID-19 syndrome overlap with the clinical features of ME/CFS.

## 1. Introduction

In March 2020, the severe acute respiratory syndrome coronavirus 2 (SARS-CoV-2) was declared a global pandemic by the World Health Organization. Mexico has been one of the worst affected countries with over 2,319,519 confirmed cases and 214,095 deaths related to SARS-CoV-2 until 23 April 2021. The epidemiological, clinical characteristics, pathogenesis, and complications of patients with COVID-19 at acute phase have been well described. An increasing number of apparently cured people are being discharged from hospitals following COVID-19 infection. However, the prevalence, mechanisms, nature and duration of symptoms in convalescent survivors remain largely unknown. There has been recent interest in the so-called post-acute sequelae of SARS-CoV-2 syndrome, long COVID, long-haul post-COVID-19, post-COVID fatigue, viral syndrome post-COVID-19, post-acute COVID-19, post-COVID-19 neurological syndrome, or post-COVID-19 syndrome, whereby patients have persistence of a multitude of symptoms following initial infection resolution. Only a few studies have systematically investigated the symptoms in the recovery of COVID-19 survivors, but the dynamic changes of these remain largely unknown [1,2,3,4,5,6].

Furthermore, it has not yet been well established if gender, age, underlying diseases, viral load, or duration of COVID-19 significantly increase the risk of developing long-term effects of COVID-19. Series have reported the incidence of persistent symptoms ranging from 40% to 90% of patients, but the interpretation of results is hampered by non-systematic and short-term evaluations, with high heterogeneity in relation to age, severity of infection, follow-up, and characteristics of the clinical evaluation [4].

Severe acute respiratory syndrome (SARS) and Middle East respiratory syndrome (MERS) are two other major coronavirus disease outbreaks that have occurred in the 21st century. Alongside acute challenges, the SARS and MERS outbreaks resulted in a chronic post-viral syndrome characterized by chronic fatigue, nonspecific myalgia, depression, sleep disturbances, and reduced ability to exercise in some survivors up to 6 months after discharge from hospital [7]. To date, it remains unknown whether and to what extent symptom burden in post-COVID-19 is comparable with symptom burden in other post-infection syndromes.

Persistent symptoms in hospitalized and non-hospitalized patients with COVID-19, regardless of severity of initial infection, have been observed at 3 and 6 months after a SARS-CoV-2 infection [6,8,9]. Fatigue is the most common symptom of acute and long COVID-19, which is not associated with severity of initial infection, although the underlying mechanisms are unclear [9]. It is present even after 6 months of the first symptom of acute COVID-19 with a prevalence of 58–63% [10]. Similar to other post-viral/ infectious syndromes, COVID-19 appears to result in symptoms that outlast the initial acute illness [11,12]. Symptoms observed in post COVID-19 patients resemble Myalgic Encephalomyelitis/Chronic Fatigue Syndrome (ME/CFS), a complex, controversial, common, and debilitating clinical condition of unknown origin, which has largely been diagnosed using consensus-based case definitions [13,14,15,16,17]. An international online survey of 3762 patients who, by self-report, experienced symptoms consistent with COVID-19, showed at 7 months after the onset of the illness that 77.9% remained with fatigue [18]. Townsed et al. studied 128 confirmed COVID-19 patients and found that, at a median of 10 weeks after the initial COVID-19 symptoms, 52% reported persistent fatigue and 31% had not returned to work [9].

Even though the exact mechanism that underlies ME/CFS has not been fully elucidated, evidence for a somatic origin of the disease is accumulating. ME/CFS often starts after an infection. It has been suggested that the infection initiates in genetically predisposed individuals to a chronic auto-reactive process, which affects several functions, including brain and energy metabolism. Recently, the U.S. National Academies of Sciences, Engineering and Medicine recommended a new name for ME/CFS, “systemic exhaustion intolerance disease” (SEID) [15]. The case definition of ME/CFS requires that the illness must have lasted for at least 6 months.

Studying the long-term symptoms of COVID-19 is critical for understanding the full natural history of disease and accurately predicting the cumulative impact of disease beyond hospitalization and mortality. The reported data does not allow determining how many patients with COVID-19 meet the ME/CFS criteria, but it is plausible that in the future as the number of infected people increases, we will see substantial growth in the number of cases with ME/CFS [19,20]. Perrin et al. [21] proposed that, as happened after the SARS outbreak, a proportion of COVID-19 affected patients might develop a severe post-COVID-19 syndrome characterized by long-term symptoms resembling ME/CFS.

COVID-19 not only threatens people’s physical health but also could affect their mental health. Casagrande et al., conducted an online survey in 2291 patients, 57.1% of participants reported poor sleep quality, 32.1% high anxiety, 41.8% high distress, and 7.6% post-traumatic stress disorder linked to COVID-19 [22]. Moreover, Cortés-Alvarez et al. published an online survey of 1105 individuals from 32 states in Mexico; 15.7% reported moderate-severe depressive symptoms, 22.6% reported moderate-severe anxiety symptoms, and 19.8% reported moderate-severe stress levels [23].

The primary aim of this study was to describe the prevalence, nature, evolution and potential risk factors of symptoms in patients discharged to home after hospitalization for moderate to severe COVID-19. For the purpose of this study, we defined post-acute COVID-19 as persistent symptoms beyond 12 weeks from the onset of COVID-19 infection [24]. Secondarily, we tried to determine in this cohort how many individuals met the case definition of ME/CFS for at least 6 months. We also sought to investigate whether there was a relationship between fatigue and a variety of clinicopathological parameters.

## 2. Materials and Methods

### 2.1. Study Cohort

This single-center prospective longitudinal study was carried out in our SARS-CoV-2 referral university hospital in Mexico City, Mexico. We enrolled consecutive adult patients hospitalized with moderate to severe confirmed COVID-19 pneumonia at hospital admission. Patients were classified according to the World Health Organization (WHO) guidelines; moderate disease was defined as clinical signs of pneumonia, such as fever, cough, dyspnea, and/or tachypnea, but no signs of severe pneumonia and, in particular, an oxygen saturation (SaO2) ≥ 90% on room air; severe disease was defined as clinical signs of pneumonia plus one of the following: respiratory rate >30 breaths/min, severe respiratory distress, or SaO2 < 90% on room air [25]. A total of 225 patients were admitted with a confirmed diagnosis from 11 April 2020 to 15 September 2020, and 160 were cured and discharged from the hospital by 22 September 2020. Thus, the time frame from the discharge of the hospital to the follow-up completion on 22 March 2021 was 6 months. All patients included in this cohort had a positive real-time reverse transcription-polymerase chain reaction (PCR) test, routine blood workup (including complete blood count, inflammatory markers, and metabolic panel). Diagnosis of COVID-19 pneumonia was performed by chest computed tomography scan in 96 (60%) patients or chest x-ray in 64 (40%) patients. All of them required supplemental oxygen. Twenty-nine patients (22%) required mechanical ventilation in the intensive care unit (ICU) with a mean of 15 ± 11 days of respiratory support.

Since fatigue is recognized as one of the most common presenting symptoms in individuals infected with SARS-CoV-2 and given that concerns have been raised that it has the potential to trigger a post-viral fatigue syndrome, we categorized our participants according to the presence or absence of fatigue. It was defined in accordance with the ME/CFS criteria (a symptom which is not the result of ongoing exertion, is not relieved by rest, occurs after minimal physical or mental/cognitive exertion and is medically unexplained) [26].

An informed consent was waived due to the minimal risk characteristics of an observational study.

### 2.2. Data Collection

Data regarding demographic, clinical characteristics, pre-existing relevant comorbidities considered to confer high risk for severe COVID-19, and laboratory findings were retrieved from the electronic medical records. We excluded the following patients: 1. Those who died before the follow-up started, 2. Those who declined to participate, 3. Those unable to be contacted. The remaining 130 survivors were contacted by phone by trained physicians and were asked to answer a previously designed questionnaire with a checklist of symptoms. Participants were asked a binary question regarding symptoms within six possible ME/CFS domains by organ system, according to the International Consensus Criteria published in 2011 [14]: 1. Fatigue and dyspnea (manifested at rest or post exertional), 2. Neurocognitive and neurosensory dysfunction, (concentration impairment, short-term memory loss), 3. Sleep dysfunction (sleeping disturbances, unrefreshing sleep), 4. Autonomic dysregulation (postural dizziness, lightheadedness, tachycardia, chest pain, temperature intolerance, sweating alterations, gastrointestinal, and genitourinary disturbances), 5. Pain disturbances (headache, joint and muscle pain), and 6. Psychiatric (anxiety, depression), in comparison to their pre-COVID-19 (baseline) status. Any remaining or newly occurring symptoms at 3 and 6 months after discharge from the hospital were recorded. The survivor’s current symptoms were distinguished from those of their pre-COVID-19 acute phase. The course of each symptom during follow-up was documented in detail.

### 2.3. Statistical Analysis

Descriptive statistical analysis was performed for all variables. Continuous variables were presented as mean (standard deviation, SD) or median (interquartile range, IQR), and dichotomous variables were expressed as absolute frequencies (percentage). Group comparisons were performed using Student’s *t*-test or Mann–Whitney U test for continuous variables and the Chi-squared test or Fisher’s exact test for categorical variables. To investigate the impact of individual variables on the presence of residual fatigue after hospitalization, participants were categorized into two groups according to fatigue. To compare fatigue at 3 months and 6 months we used Chi-squared test or Fisher’s exact test for categorical variables. Demographic data, comorbidities, body mass index (BMI), clinical data, and laboratory values at admission were included as predictors of fatigue. Multivariable adjusted logistic regression models were used to estimate the odds ratios (ORs) and 95% confidence intervals (CIs) for association between fatigue and risk factors. All tests were two-sided, and a *p*-value less than 0.05 was considered statistically significant. We included all participants for whom the variables of interest were available in the final analysis, without missing data. All statistical analyses were done with SPSS v. 15.

## 3. Results

### 3.1. Study Population

**Figure 1:**
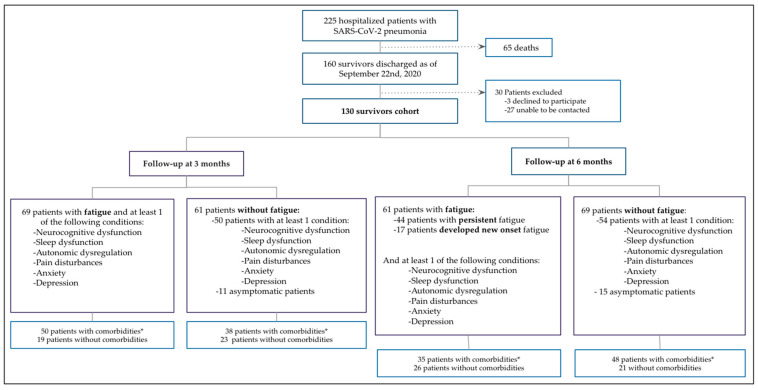
Flow Chart of the Study Design. * Comorbidities: Hypertension, Diabetes, Chronic obstructive pulmonary disease, Chronic Heart disease, Chronic Kidney disease, Immunosuppressive condition or Psychiatric disorder.

During the study period, a total of 767 consecutive patients were evaluated in the dedicated triage unit for suspected SARS-CoV-2 infection; of them, 225 (29.3%) were admitted with a diagnosis of confirmed SARS-CoV-2 pneumonia (a detailed flowchart of the study design is shown in Figure 1. Sixty-five patients (28.8%) died during hospitalization and 160 (71.1%) were discharged after recovery and eligible for the follow-up post-acute care assessment. Three patients (1.8%) refused to participate and 27 (16.8%) could not be contacted. Finally, 130 patients were successfully followed up and considered as the study cohort.

### 3.2. Demographics and Clinical Characteristics

Baseline characteristics are summarized in Table 1. The mean age of the 130 survivors was 51 ± 14 years, ranging from 18 to 80 years, 45 (34.6%) were female. The most common comorbidity was hypertension in 53 (41%) patients, followed by type 2 diabetes in 41 (32%), and obesity in 38 (29%). The mean duration of hospital stay was 15 ± 12 days. Laboratory findings at admission are presented in Table 1. Overall, inflammatory biomarkers were elevated. Patients acutely infected with SARS-CoV-2 demonstrated decreased lymphocyte counts, higher neutrophil counts, elevated C-reactive protein (CRP), ferritin, D-dimer, as well as pro-inflammatory cytokines such as interleukin-6 (IL-6) and vascular endothelial growth factor (VEGF).

Three months after discharge from the hospital, 119 (91.5%) patients had persistence of at least one symptom that did not exist before COVID-19 with a median of 9 (IQR 4–13) symptoms and at 6 months of follow-up, 115 (88%) patients reported at least one symptom with a median of 9 (IQR 5–14) symptoms.

As expected, fatigue was the most frequently persistent symptom at 3 months follow-up, reported by 69 (53%) patients. There were some significant demographic differences in those patients with or without fatigue. Patients with fatigue at three months follow-up were older, 53.9 ± 13.5 years compared with 48.5 ± 13.3 years in those without fatigue (*p* = 0.02) and had a longer length of hospital stay 17 ± 14 days vs. 13 ± 10 days (*p* = 0.04), although their distribution by gender was similar. Twenty-eight (40.5%) patients with fatigue were females compared with 17 (27.8%) without fatigue (*p* = 0.09). There was no difference in BMI between patients with fatigue and those without it. Overall, the remaining most prevalent symptoms at three months follow-up were: dyspnea on effort 66 (50.8%), short-term memory loss 59 (45.4%), sleep disturbances 59 (45.4%), myalgias 53 (40.8%), tachycardia 53 (40.8%), postural dizziness 51 (39.2%), anxiety 51 (39.2%), depression 46 (35.3%), and joint pain in 50 (38.5%) patients (Table 2). The rate of post-COVID fatigue decreased over time since only 61 (46.9%) patients reported this symptom after 6 months.

As shown in Table 2, on univariate analysis of differences in those with and without fatigue at three months follow-up, there was a significantly higher prevalence of symptoms in those with fatigue: dyspnea in 44 (63.8%), tachycardia in 36 (52.2%), sleep disturbances in 41 (59.4%), postural dizziness in 31 (44.9%), constipation in 35 (50.7%), muscle pain in 39 (56.5%) and joint pain in 34 (49.3%), all of them with high statistical significance. In patients with persistent fatigue at six months follow-up, a significantly higher prevalence of the majority of symptoms was also reported.

There is a large discrepancy in the prevalence of ME/CFS as a result of using different definitions. The more specific the exclusion criteria in the definition, the smaller the number of patients diagnosed with ME/CFS. Based on the original criteria of ME/CFS proposed by the Centers for Disease Control and Prevention (CDC) Fukuda definition of 1994 [13], the only mandatory feature of ME/CFS is unexplained chronic fatigue, which must be accompanied by at least four out of eight minor symptoms related to neurological, cognitive, sleep, autonomic, gastrointestinal, and genitourinary disturbances, plus pain. In our survey, 23 (17.6%) patients met this case definition criteria. The Canadian clinical case definition [14] specifies that post exertional malaise (a vague feeling of discomfort or fatigue) must occur with a loss of physical or mental stamina, muscle, or cognitive fatigability. In addition, there need to be two or more neurological/cognitive manifestations, (unrefreshing sleep or poor sleep quality), as well as a significant degree of arthralgia and/or myalgia. Finally, there needs to be at least one symptom from two of the following categories: autonomic manifestations (neurally mediated hypotension, postural orthostatic tachycardia, lightheadedness, palpitations with or without arrhythmias), neuroendocrine manifestations (recurrent feelings of feverishness and cold extremities), and immune manifestations (recurrent sore throats). Therefore, in our cohort, 20 (15.3%) patients met these criteria. Compared with the International Consensus Criteria of 2011 [15], where a patient has to report at least eight (one mandatory: post-exertional neuroimmune exhaustion, and seven variable symptoms) to meet the diagnosis of ME, while autonomic, sensory and cognitive dysfunctions are not compulsory; 25 (19.2%) of our patients met the diagnosis criteria. The Institute of Medicine [16], recently proposed a new case definition that included the following 4 symptoms: substantial reduction or impairment in the ability to engage in pre-illness levels of occupational, educational, social, or personal activities; post-exertional malaise, unrefreshing sleep; and at least one of the two following symptoms: cognitive impairment or orthostatic intolerance. According to this definition, the presence of other illnesses should not preclude patients from receiving a diagnosis of ME/CFS (SEID) except in the event that all the symptoms can be accounted for by these other illnesses. Following the SEID case definition in our cohort, only 17 (13%) patients met the criteria for ME/CFS.

**Figure 2:**
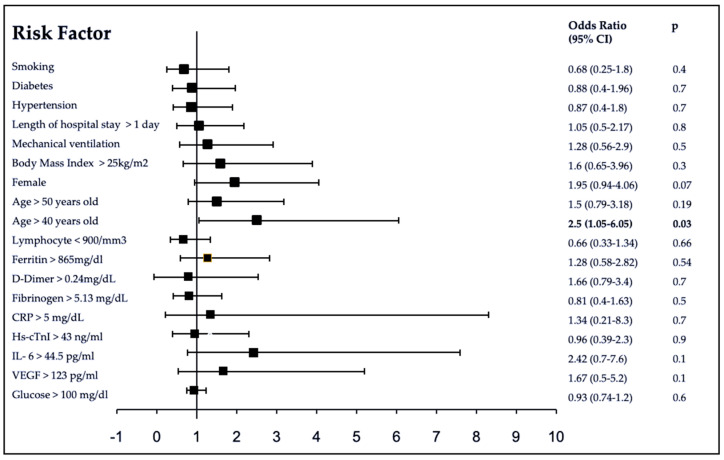
Associated factors in Chronic Fatigue in Post COVID-19. The 95% confidence intervals (CI) of the odds ratios have been adjusted for multiple testing. In bold, independent predictors associated with the outcomes. To the purpose of logistic regression, variables were categorized regarding their 75-percentiles or lowest levels where 25-percentiles were used of our population. C-Reactive Protein (CRP), High sensitive cardiac Troponin I (hs-cTnI), Interleukin-6 (IL-6), Vascular Endothelial Growth Factor (VEGF).

After multivariate adjustment of all baseline clinical features and laboratory biomarkers, only age 40 to 50 years old was positively associated with fatigue with an OR 2.5 (95% CI 1.05–6.05) *p* = 0.03. A risk trend towards the female sex was observed, however it did not reach statistical significance, OR 1.95 (0.94–4.06) *p* = 0.07 (Figure 2).

## 4. Discussion

In our study of 130 patients who were evaluated 3 and 6 months after hospitalization for COVID-19, 91.5% reported at least 1 symptom that did not exist before the infection. In a recent systematic review to assess the frequency and variety of persistent symptoms among patients with COVID-19, based on PubMed and Web of Science search, 16 studies, most of which comprised patients who were previously hospitalized, reported the persistence of at least 1 symptom with a median frequency of 72.5% (IQR, 55.0–80.0%) [27]. We found that the most common symptoms in our patients were fatigue, new-onset dyspnea, sleeping and neurocognitive disturbances, orthostatic intolerance, anxiety, depression, and muscle and joint pain.

We observed fatigue in 69 (53%) of COVID-19 survivors. Nasserie et al. [27], reported that fatigue was frequently experienced by patients with persistent symptoms after COVID-19 (median frequency 40.0%, IQR, 31.0–57.0%). In our survey, patients with fatigue were older, and persistent fatigue at 3 and 6 months was significantly associated with ages 40 to 50 years old. This finding is comparable with the systematic review by Nasserie et al. [27] where 30 studies reported mean or median age younger than 60 years, and 14 studies reported mean or median ages of 50 years or younger. Carvalho-Schneider et al. [5] described the clinical evolution and predictors of symptom persistence during 2 months of follow-up in adults with noncritical COVID-19, and persistent symptoms at 60 days were significantly associated with ages 40 to 60 years old.

All the other baseline characteristics (including gender, comorbidities, and laboratory studies) of patients in our cohort showed no statistical difference in those with persistent fatigue compared with those who did not have it.

The available data regarding the incidence and evolution of post COVID symptoms are scarce and heterogeneous. In Italy, Carfi et al. [1] reported persistence of symptoms in 87.4% of 143 patients discharged from hospital who recovered from acute COVID-19 at a mean follow-up of 60 days from the onset of the first symptom. Fatigue (53.1%), dyspnea (43.4%), joint pain (27.3%), and chest pain (21.7%) were the most commonly reported symptoms, with 32% reporting 1 or 2 symptoms, while 55% of patients continued to experience 3 or more symptoms. Halpin et al. [4] in the United Kingdom reported the results of a structured telephone interview in 100 patients with a mean of 48 days post discharge from hospital, their findings were similar to ours, fatigue was the most commonly reported symptom by 72% of participants. The next most common symptoms were breathlessness (65.6%) and psychological distress (46.9%).

Although psychological distress was not examined in our study with a standardized scale or questionnaire, the frequencies of depression and anxiety were higher than those reported by other studies (35.3% and 39.2% respectively), while in other Mexican studies, depression was found in 15.7% and anxiety in 22.6% of respondents [23].

An important finding in our study is that the prevalence of symptoms seems to progressively decrease over time. At 6 months post-discharge interview, fatigue declined from 69 (53%) to 61 (46.9%) patients. Wang et al. [28] evaluated 131 COVID-19 patients (median age 49) weekly for up to 4 weeks. At discharge, 40.4% had symptoms, which progressively declined to 9.1% after 4 weeks.

Fatigue is a multidimensional health problem. As demonstrated in our study, those patients with fatigue had a higher prevalence of other symptoms such as breathlessness, cognitive dysfunction, sleep disturbances, autonomic dysregulation, and psychological distress. The rates of post-COVID fatigue in this and previous studies appear to be higher than those previously reported following other viral infections at a similar interval time [29]. Some of the symptoms of post-COVID syndrome, such as fatigue, myalgia, cognitive disturbances, unrefreshing sleep, orthostatic intolerance, exaggerated postural tachycardia, and hyperadrenergic surges are suggestive of autonomic dysfunction such as those seen in ME/CFS. Although these symptoms parallel those that are seen in post-infectious ME/CFS, data supporting COVID-19 as an infectious trigger for ME/CFS are limited. An observational study investigating post-acute-COVID-19 symptoms as defined by ME/CFS criteria does not yet exist [30]. To date, only a few case reports of confirmed or probable ME/CFS after COVID-19 have been published [31]. Orthostatic intolerance is a hallmark clinical characteristic of ME/CFS. Most symptoms of orthostatic intolerance are related to a reduced cerebral blood flow [32]. Novak [33], described a post COVID-19 patient who developed chronic fatigue, orthostatic dizziness, and brain fog consistent with orthostatic hypoperfusion syndrome (OCHOS), a form of orthostatic intolerance. Dani et al. [34] described a series of individuals with symptoms of long COVID with orthostatic intolerance. In our study, 39.2% of patients developed after hospitalization orthostatic dizziness and 16.9% developed lightheadedness after prolonged hospitalization.

Although less frequent compared to inflammatory and immune-mediated disorders, infectious diseases may also affect the autonomic nervous system. There are several pathogenic mechanisms that explain why infections can induce autonomic dysfunction, including the direct invasion of the central nervous system resulting in increased central sympathetic outflow, the involvement of the peripheral autonomic system, and a toxin-mediated effect. Immune-mediated mechanism occurring during or after an infection is a well-known trigger of some autonomic neuropathies. SARS-CoV-2 might also infect and destroy extra cardiac postganglionic sympathetic noradrenergic neurons resulting in splanchnic venous pooling or a failure of mesenteric vasoconstriction during orthostasis, secondarily increasing cardiac sympathetic noradrenergic efferent traffic with postural tachycardia syndrome [35]. It has been well established that autonomic disorders, such as ME/CFS and postural orthostatic tachycardia syndrome are associated with autoantibodies to ß2 adrenoceptors, which could cause ß2 adrenergic receptor dysfunction and the consequent involvement in their pathophysiology [36]. Recently, novel hypotheses have proposed virus-induced alterations in mitochondrial metabolism [37] and autoimmune systems [38]. These hypotheses are related to some pathophysiologic features involving impairment on the central and autonomic nervous system, metabolic function and the immune system [39].

ME/CFS has been associated with viral infections so COVID-19 patients could also develop this illness. Since the first recognition of ME/CFS in a poliomyelitis outbreak in 1934, the illness has undergone various changes in terminology and case definition. Nowadays, the diagnoses of ME and CFS are based upon subjective symptoms reported by patients. According to the published case definitions of ME/CFS, we allocated the most prominent definitions into categories based on the symptoms’ characteristics. It was shown in our study that 17.6% met the commonly used Fukuda criteria for ME/CFS [13], while only 13% fulfill the more stringent criteria proposed by the Institute of Medicine [16].

“Long COVID-19” was first used as a Twitter hashtag to describe persistent symptoms after acute COVID-19; following intense advocacy by patients across the world, the WHO adopted this patient-coined term. The so-called long COVID-19 or if preferred post-acute COVID-19 syndrome may well include several conditions that have more than one etiology and may last a long time in the same patient.

### Limitations

The study has several limitations. It was conducted in a relatively small sample size at a tertiary single care center, with telephonic interviews using a binary questionnaire. Therefore, our results could not be accurate to diagnose ME/CFS. This investigation was an uncontrolled cohort study, which precludes any comparison of findings with individuals not experiencing COVID-19. Several patients who were asked to participate declined telephone surveys: it is possible that patients who did not participate were less symptomatic than those who did. The findings are also limited by the lack of imaging studies to evaluate residual lung, myocardial or brain involvement which could explain the symptoms.

## 5. Conclusions

The current study represents, to our knowledge, a novel report in literature examining the potential relationship between the symptoms of post-acute COVID-19 Syndrome and ME/CFS in a large cohort.

This study demonstrated that significant abnormalities still exist in a high proportion of COVID-19 patients up to 6 months after discharge. It is necessary to follow-up these patients, performing comprehensive assessment and early rehabilitation for detection and appropriate management of persistent or emerging long-term symptoms. The full range of the duration and severity of post-acute COVID-19 is currently unknown. A consensus is needed regarding when and how to classify manifestations in the post-acute period, considering many of these symptoms may resolve with time and their prevalence, therefore, depends on the time of evaluation [40]. Longer longitudinal studies and further research would be required to understand the mechanisms underlying long-term COVID-19 symptoms and their precise connection with ME/CFS.

## Figures and Tables

**Table 1:** Demographic characteristics of study participants.

Characteristic	Overall (*n* = 130)	Non-Fatigue (*n* = 61)	Fatigue (*n* = 69)	*p*-Value
Age (years)	51.0 ± 14	48.5 ± 13.2	53.0 ± 13.5	0.02
Female *n* (%)	45 (34.6)	17 (27.8)	28 (40.5)	0.09
Active smoker *n* (%)	23 (17.6)	12 (19.6)	11 (15.6)	0.3
Body Mass Index (kg/m^2^)	26.6 ± 3	27.6 ± 3.8	28.2 ± 5.1	0.5
Underlying Diseases *n* (%)				
Hypertension	53 (40.7)	22 (36.0)	31 (44.9)	0.19
Overweight	62 (47.6)	35 (57.3)	27 (39.1)	0.11
Obesity	38 (29.2)	13 (21.3)	25 (36.2)	0.10
Diabetes	41 (31.5)	17 (27.8)	24 (34.7)	0.25
Chronic heart disease	22 (16.9)	9 (14.7)	13 (18.8)	0.35
Chronic obstructive pulmonary disease	4 (3.0)	1 (1.6)	3 (4.3)	0.35
Chronic kidney disease	14 (10.7)	5 (8.1)	9 (13.0)	0.27
Immunosuppressive condition	15 (11.5)	7 (11.4)	8 (11.5)	0.6
Psychiatric disorder	2 (1.5)	0	2 (2.8)	0.28
Acute COVID-19 Characteristics				
Mechanical ventilation *n* (%)	29 (22.3)	12 (19.6)	17 (24.6)	0.32
Days on mechanical ventilation	15 ± 11	14 ± 10.5	15 ± 12	0.83
Length of hospital stay in days	15 ±12	13 ± 10	17 ± 14	0.04
Days of follow-up since discharge	270 ± 32	266 ± 30	273 ± 36	0.8
Laboratory Tests Median (IQR)				
Ferritin ng/mL	512 (200–865)	501 (255–820)	520 (162–880)	0.73
NT-proBNP pg/mL	242 (89–1055)	200 (86–803)	313 (94–1803)	0.23
Hs-cTnI pg/mL	9.2 (4.7–23)	6.8 (4.7–18.5)	11.4 (4.8–25.4)	0.26
D-Dimer ng/mL	0.35 (0.18–0.56)	0.34 (0.16–0.76)	0.37 (0.19–0.55)	0.37
CRP mg/L	121 (53.7–216.5)	121 (57.8–217)	123.5 (42.5–218.7)	0.86
Fibrinogen g/L	5.26 (4.49–6)	5.5 (4.8–6)	5.09 (4.3–6)	0.16
IL-6 pg/mL	4.5 (4.5–44.5)	7.9 (4.5–64)	4.5 (4.5–39.5)	0.63
VEGF pg/mL	53.2 (15–123.1)	46.6 (15–119)	60.6 (15–129.6)	0.74
Neutrophil count, 10^3^/L	6.7 (4.1–10.5)	6.7 (4.5–11.6)	6.8 (4.1–9)	0.30
Lymphocyte count, 10^3^/L	0.8 (0.6–1.2)	0.9 (0.6–1.2)	0.8 (0.6–1.2)	0.83
AST U/L	36 (22.3–57.9)	36 (22.2–58.7)	37.2 (22.1–56.5)	0.80
Glucose mg/dL	122 (105–153)	120 (105.4–153)	125 (104–153)	0.81
Creatinine mg/dL	0.9 (0.73–1.2)	0.9 (0.72–1.24)	0.9 (0.73–1.28)	0.69

Data are presented as *n* (%) or median (Interquartile range): *p* values were calculated by *t*-student, Chi-square, Mann-Whitney U-test. High sensitive cardiac Troponin I (hs-cTnI), C-Reactive Protein (CRP), Interleukin-6 (IL-6), Vascular Endothelial Growth Factor (VEGF), Aspartate Aminotransferase (AST).

**Table 2:** Post-acute COVID-19 symptoms.

Symptoms	Overall (*n* = 130)	Non-Fatigue (*n* = 61)	Fatigue (*n* = 69)	*p*-Value	Overall (*n* = 130)	Non-Fatigue (*n* = 69)	Fatigue (*n* = 61)	Non-Fatigue at 3 Months vs. Non-Fatigue at 6 Months *p*-Value	*p*-Value	Fatigue at 3 Months vs. Fatigue at 6 Months *p*-Value
Fatigue	69 (53.0)	0	69 (100)	<0.001	61 (46.9)	0	61 (100)	<0.001	0.3	0.19
**Respiratory**										
Resting dyspnea	21 (16.2)	6 (9.8)	15 (21.7)	0.053	21 (16.2)	4 (5.8)	17 (27.9)	0.001	0.29	0.34
Dyspnea on effort	66 (50.8)	21 (34.4)	44 (63.8)	0.001	55 (42.3)	16(23.2)	39 (63.9)	<0.001	0.11	0.5
**Neurocognitive, Neurosensory, Perceptual Disturbances**										
Concentration impairment	30 (23.1)	8 (13.1)	22 (31.9)	0.009	40 (30.8)	10 (14.5)	30 (49.2)	<0.001	0.51	0.05
Short-term memory loss	59 (45.4)	17 (27.9)	36 (52.2)	0.004	70 (53.8)	29 (42)	41 (67.2)	0.003	0.06	0.08
Inability to focus vision	50 (38.5)	18 (29.5)	34 (49.3)	0.017	43 (33.1)	13 (18.8)	30 (49.2)	<0.001	0.11	0.56
Light sensitivity	24 (18.5)	4 (6.6)	17 (24.6)	0.004	26 (20.0)	8 (11.6)	18 (29.5)	0.01	0.24	0.33
Anosmia	16 (12.3)	3 (4.9)	11 (15.9)	0.039	9 (6.9)	4 (5.8)	5 (8.2)	0.42	0.56	0.14
Ageusia	16 (12.3)	1 (1.6)	13 (18.8)	0.001	7 (5.4)	3 (4.3)	4 (6.6)	0.43	0.35	0.03
Tingling	61 (46.9)	20 (32.8)	38 (55.1)	0.009	61 (46.9)	26 (37.7)	35 (57.4)	0.019	0.34	0.54
**Sleep Disturbances**										
Disturbance of sleep	59 (45.4)	17 (27.9)	41 (59.4)	<0.001	66 (50.8)	28 (40.6)	38 (62.3)	0.011	0.09	0.43
Unrefreshin sleep	50 (38.4)	15 (24.6)	31 (44.9)	0.012	63 (48.5)	25 (36.2)	38 (62.3)	0.003	0.1	0.03
**Autonomic Dysregulation**										
Postural dizziness	51 (39.2)	16 (26.2)	31 (44.9)	0.021	46 (35.4)	13 (18.8)	33 (54.1)	<0.001	0.21	0.24
Lightheadedness when prolonged standing	22 (16.9)	7 (11.5)	15 (21.7)	0.092	22 (16.9)	7 (10.1)	15 (24.6)	0.025	0.51	0.51
Chest pain	40 (30.8)	9 (14.8)	25 (36.2)	0.004	36 (27.7)	9 (13)	27 (44.3)	<0.001	0.48	0.28
Tachycardia	53 (40.8)	15 (24.6)	36 (52.2)	0.001	46 (35.4)	17 (24.6)	29 (47.5)	0.005	0.57	0.36
Change pattern of sweating	47 (36.2)	17 (27.9)	26 (37.7)	0.159	37 (28.5)	11 (15.9)	26 (42.6)	0.001	0.07	0.34
Intolerance to temperature	41 (31.5)	12 (19.7)	28 (40.6)	0.008	40 (30.8)	12 (17.4)	28 (45.9)	<0.001	0.45	0.33
**Gastrointestinal and Genitourinary**										
Stomach bloated after meals	35 (26.9)	9 (14.8)	24 (34.8)	0.007	45 (34.6)	12 (17.4)	33 (54.1)	<0.001	0.43	0.03
Abdominal pain	20 (15.4)	5 (8.2)	13 (18.8)	0.065	20 (15.4)	3 (4.3)	17 (27.8)	<0.001	0.29	0.15
Diarrhea	19 (14.6)	6 (9.8)	13 (18.8)	0.114	19 (14.6)	6 (8.7)	13 (21.3)	0.03	0.62	0.44
Constipation	48 (36.9)	14 (22.9)	35 (50.7)	0.001	36 (27.7)	12 (17.4)	24 (39.3)	0.005	0.28	0.13
Nausea	19 (14.6)	3 (4.9)	16 (23.2)	0.003	22 (16.9)	5 (7.2)	17 (27.8)	<0.002	0.43	0.42
Urinary frequency	44 (33.8)	8 (13.1)	21 (30.4)	0.015	30 (23.1)	12 (17.4)	18 (29.5)	0.07	0.33	0.45
Difficulty emptying bladder	18 (13.8)	6 (9.8)	14(20.3)	0.07	14 (10.7)	4 (5.8)	10 (16.4)	0.02	0.29	0.36
Difficulty with sexual function	28 (21.5)	9 (14.8)	17 (24.6)	0.117	18 (13.8)	8 (11.6)	10 (16.4)	0.29	0.39	0.17
**Pain**										
Headache	38 (29.2)	12 (19.7)	25 (36.2)	0.028	48 (36.9)	12 (17.4)	36 (59)	<0.001	0.45	0.01
Muscle pain	53 (40.8)	14 (22.9)	39 (56.5)	<0.001	47 (36.2)	11 (15.9)	36 (59)	<0.001	0.21	0.45
Joint pain	50 (38.5)	11 (18)	34 (49.3)	<0.001	57 (43.8)	18 (26.1)	39 (63.9)	<0.001	0.18	0.06
**Psychiatric**										
Anxiety	51 (39.2)	15 (24.6)	39 (56.3)	<0.001	46 (35.4)	11 (15.9)	35 (57.4)	<0.001	0.15	0.53
Depression	46 (35.3)	13 (21.3)	31 (44.9)	0.004	45 (34.6)	13 (18.8)	32 (52.5)	<0.001	0.44	0.24

Data are presented as *n* (%), *p* values were calculated by chi-square.

## Data Availability

This study did not report any data. The study was conducted in accordance with the Declaration of Helsinki.
